# Palliative treatment of painful bone metastases with MR imaging–guided focused ultrasound surgery: a two-centre study

**DOI:** 10.1186/2050-5736-3-S1-O51

**Published:** 2015-06-30

**Authors:** Fulvio Zaccagna, Brachetti Giulia, Alberto Bazzocchi, Paolo Spinnato, Ugo Albisinni, Alessandro Napoli, Carlo Catalano

**Affiliations:** 1 University of Rome – Sapienza, Rome, Italy; 2 The “Rizzoli” Orthopaedic Institute, Bologna, Italy

## Background/introduction

To evaluate the efficacy of non-invasive high intensity MR guided focused Ultrasound Surgery (MRgFUS) for pain palliation of bone metastasis in patients who had exhausted EBRT or refused other therapeutic options.

## Methods

This is a prospective, single arm, multicentre study performed after IRB approval. 72 patients (female: 24, male: 48, mean age: 61.6) with painful bone metastases were enrolled. 87 non-spinal lesions underwent MRgFUS treatment using ExAblate 2100 system (InSightec). European Organization for Research and Treatment of Cancer QLQ-BM22 was used for clinical assessment additionally to Visual Analog Scale (VAS), at baseline and 1,3 and 6 months after treatment. All patients underwent CT and MRI before treatment and 3-6 months afterward.

## Results and conclusions

Results: No treatment-related adverse events were recorded. 34/72 (47.2%) patients reported complete response to treatment and discontinued medications. 29/72 (40.3%) experienced a pain score reduction >2 points, consistent with partial response. Remaining 9 (12.5%) patients had recurrence after treatment. Statistically significant differences between baseline (6, 95%CI 5-8) and follow-up (2, 95%CI 0-3) VAS values and medication intake were observed (p<0.05). Similarly a significant difference was found for QLQ-BM22 between baseline and follow-up (p<0.05).

Conclusion: MRgFUS can be safely and effectively used as totally noninvasive treatment for pain palliation of bone metastasis in patients who had exhausted EBRT and also in patients not previously treated with EBRT.

